# The Use of High Surface Area Mesoporous-Activated Carbon from Longan Seed Biomass for Increasing Capacity and Kinetics of Methylene Blue Adsorption from Aqueous Solution

**DOI:** 10.3390/molecules26216521

**Published:** 2021-10-28

**Authors:** Panuwat Lawtae, Chaiyot Tangsathitkulchai

**Affiliations:** Institute of Engineering, School of Chemical Engineering, Suranaree University of Technology, Muang District, Nakhon Ratchasima 30000, Thailand; pnwlawtae@gmail.com

**Keywords:** adsorption capacity, adsorption kinetics, adsorption isotherms, activated carbon, longan fruit seed, methylene blue

## Abstract

Microporous- and mesoporous-activated carbons were produced from longan seed biomass through physical activation with CO_2_ under the same activation conditions of time and temperature. The specially prepared mesoporous carbon showed the maximum porous properties with the specific surface area of 1773 m^2^/g and mesopore volume of 0.474 cm^3^/g which accounts for 44.1% of the total pore volume. These activated carbons were utilized as porous adsorbents for the removal of methylene blue (MB) from an aqueous solution and their effectiveness was evaluated for both the adsorption kinetics and capacity. The adsorption kinetic data of MB were analyzed by the pseudo-first-order model, the pseudo-second-order model, and the pore-diffusion model equations. It was found that the adsorption kinetic behavior for all carbons tested was best described by the pseudo-second-order model. The effective pore diffusivity (D_e_) derived from the pore-diffusion model had the values of 4.657 × 10^−7^–6.014 × 10^−7^ cm^2^/s and 4.668 × 10^−7^–19.920 × 10^−7^ cm^2^/s for the microporous- and mesoporous-activated carbons, respectively. Three well-known adsorption models, namely the Langmuir, Freundlich and Redlich–Peterson equations were tested with the experimental MB adsorption isotherms, and the results showed that the Redlich–Peterson model provided the overall best fitting of the isotherm data. In addition, the maximum capacity for MB adsorption of 1000 mg/g was achieved with the mesoporous carbon having the largest surface area and pore volume. The initial pH of MB solution had virtually no effect on the adsorption capacity and removal efficiency of the methylene blue dye. Increasing temperature over the range from 35 to 55 °C increased the adsorption of methylene blue, presumably caused by the increase in the diffusion rate of methylene blue to the adsorption sites that could promote the interaction frequency between the adsorbent surface and the adsorbate molecules. Overall, the high surface area mesoporous carbon was superior to the microporous carbon in view of the adsorption kinetics and capacity, when both carbons were used for the removal of MB from an aqueous solution.

## 1. Introduction

Adsorption has gained increasing acceptance in the separation and purification processes for both gas and liquid systems, due to its process simplicity, less energy consumption, high separation efficiency at trace concentrations, low maintenance cost, and adsorbent reusability [1,2]. Among the various available commercial adsorbents, activated carbon has been recognized as the oldest and most widely used porous adsorbent for a variety of applications and is the most popularly used adsorbent in water and wastewater treatment processes [3]. This arises from its large specific surface area and pore volume, the flexibility of pore manipulation by controlling the preparation conditions, and the capability of surface chemistry modification to improve the adsorbate selectivity. Concerning the adsorption in a liquid phase, it is generally recognized that dyes are one of the most detrimental pollutants present in industrial wastewater. Dyes are natural or synthetic organic compounds that are utilized in various industries such as leather, paper, rubber, textile, plastic, cosmetic, pharmaceutical, and food industries [4]. Colored dye wastewater is generated during the production of the dye itself or as a result of its use in textile and related industries. Dyes can also affect aquatic plants and ones who use these effluents without awareness of their detrimental health effects from washing, bathing, and drinking [5]. Therefore, the search for inexpensive and effective porous solids for removal of dyes would be a worthwhile endeavor.

Researches on the adsorption of organic water pollutants by activated carbon have been carried out extensively and a voluminous literature is available, especially in the field of surface modification of activated carbon for effective water pollution control [6]. In general, activated carbon is more efficient in removing organic compounds than metals and inorganic pollutants, and continued research efforts have been made to increase the adsorption efficiency of carbon sorbents [7,8,9,10]. Obviously, the adsorption capacity of activated carbon is strongly dependent on the porous properties and surface chemistry of the carbon adsorbents that can be controlled by the judicious use of activation conditions, types of precursors, and chemical additives [11]. Although numerous results have been reported on the kinetics and sorption properties, much work remains to be performed for the better understanding of the underlying sorption mechanism [12].

In studying the adsorption mechanisms of a cationic dye that can dissociate into positively charged ions in an aqueous solution, methylene blue dye (MB) has often been used as a model cationic dye. Methylene blue is an organic chloride salt with a dark green color, having a formula C_16_H_18_ClN_3_S and molecular weight of 319.85 g/mol. Some important physical properties of MB are density of 1 g/mL at 20 °C, melting point of 100–110 °C, and solubility in water of 40 g/L at 20 °C. MB is mainly used in the production of coloring paper, cotton, silk and wool, and is also used as a chemical indicator, medicinal, and biological strain [13]. MB is a toxic dye, which can result in harmful effects on human and environmental problems. Common side effects include headache, vomiting, high blood pressure, and the eventual breakdown of red blood cells. The removal of MB from industrial wastes has been reported using various methods such as electrochemical removal, photodegradation reaction, chemical coagulation, membrane filtration, and physical adsorption methods [14,15,16]. Of these removing techniques, the application of adsorption utilizing low-cost adsorbents to remove this dye is an attractive process for the efficient removal of this dye, and has been the focal point of a number of investigations [4,14].

With regard to activated carbon, an understanding of the correlation between its porous properties, including the surface area and pore size distribution, and the adsorption capacity is essential for the selection of the most appropriate adsorbent for the effective removal and control of the dyes. Graham [17] studied the effect of pore size of activated carbon on MB adsorption capacity and found that the surface area of activated carbon available for MB adsorption was usually limited to the minimum permissible pore size of around 1.33 nm. Later, Reffas et al. [18] studied the adsorption of MB on activated carbon with a high proportion of mesopores and found that the highest adsorption uptake was achieved in the supermicropore size range of 1.4 to 2.0 nm. This finding was in agreement with the work of Benadjemia et al. [19], who confirmed that the adsorption of MB molecules mostly occurred in the wider supermicropore range when compared with the adsorption in meso-macroporous activated carbon. Recently, it has been reported that activated carbon with a high surface area of about 1438 m^2^/g and containing a micropore volume of around 80% of the total pore volume demonstrated an extremely high adsorption capacity of up to 1930 mg MB/g carbon [20].

However, to obtain both the low mass transfer resistance in pores (fast kinetics) and a high equilibrium adsorption capacity of relatively large dye molecules, activated carbon with a relatively high proportion of mesopore volume and a high surface area is obviously required [21,22,23,24]. In our previous work, we successfully prepared mesoporous-activated carbon from longan fruit seed with a high surface area (1773 m^2^/g) through a process of modified physical activation with CO_2_, which was referred to as the OTA method [25]. Therefore, the present work was aimed to study and compare the equilibrium and kinetics of methylene blue adsorption from aqueous solution by a microporous-activated carbon, and a high surface area mesoporous-activated carbon prepared from longan fruit seed. In addition, the effects of the process conditions including adsorbent dosage, temperature, and solution pH on the efficiency of MB dye uptake were also investigated.

## 2. Materials and Experimental Method

### 2.1. Materials

Fresh longan fruit seed was obtained from Saha-Prachinburi Foods Industry Ltd., a local fruit processing plant in Chiangmai province, Thailand. The raw longan seed was thoroughly rinsed with water and dried at 110 °C for 48 h, then it was crushed and sieved to obtain an average particle size of 1.70 mm (10 × 14 US mesh). The chemicals used in this study including NaCl, NaOH, HCl, and methylene blue were acquired from Carlo Erba (Carlo Erba Reagent, Cornaredo, Italy). All chemicals were of analytical grade and were used without further purification. The dye stock solution was prepared by dissolving accurately weighed MB dye powder in distilled water to obtain a solution with a MB concentration of 1000 mg/L. Then, different initial dye solutions required for the adsorption tests were prepared by diluting the stock solution in appropriate proportions.

### 2.2. Preparation of Longan Seed-Activated Carbons

Details for the preparation of longan seed-activated carbon follow those reported in our previous work [25]. In brief, the char was first prepared by carbonizing the longan seed under the flow of N_2_ at 500 °C for 90 min. Then, the derived char was further activated under the flow of CO_2_ at 850 °C for 120–240 min in a vertical tube furnace (CTF 12, Carbolite, Staffordshire, UK). This activation process, known as the two-step activation method, produced microporous-activated carbon with around 70–80% of the micropore volume. For the preparation of activated carbon with larger amounts of mesopores, about 15 g of activated carbon produced from the two-step activation process at the activation temperature and time of 850 °C and 1 h, respectively, were first oxidized in a quartz tube reactor by heating the activated carbon from room temperature in a stream of air (100 cm^3^/min) to the required oxidation temperature of 230 °C and held at this temperature for 12 h. The purpose of this step was to create additional oxygen functional groups on the carbon surfaces. Then, the oxidized carbon was heated at 950 °C for 2 h under nitrogen gas flowing at the rate of 100 cm^3^/min to remove most of the surface functional groups, thus giving increasing surface reactivity caused by chemical bond disruption. After that, the sample was activated again with CO_2_ at 850 °C for another 1 h. This process completed the first cycle of the preparation method for mesoporous carbon production which is referred to as the OTA method (Oxidation, Thermal destruction, and Activation). In this study, a total of three cycles for the OTA method were performed. The derived microporous carbon prepared by the conventional two-step activation was designated as A850–X, where A850 represents the activated carbon derived under the activation temperature of 850 °C and symbol X represents the activation time (120–240 min). The mesoporous carbon was designated as A850–X–Y, where A850 stands for the mesoporous- activated carbon originally prepared by activating the char at the temperature of 850 °C for time X (60 min) and Y is the number of repeated OTA cycles (1–3). The final yield of activated carbon was calculated based on the weight of the initial longan seed char.

### 2.3. Characterization of the Prepared Activated Carbons

The prepared microporous- and mesoporous-activated carbons were characterized for their porous properties using N_2_ adsorption/desorption isotherms measured at −196 °C (77 K) by using a high-performance adsorption analyzer (ASAP2010, Micromeritics, Norcross, GA, USA). The isotherms of N_2_ adsorption were shown in Figure S1 of the Supplementary Materials for the microporous- and mesoporous-activated carbons. Discussion on the N_2_ isotherm behavior of the prepared activated carbons has been presented elsewhere [25]. The specific surface area was calculated from the N_2_ adsorption isotherm data by applying the Brunauer–Emmett–Teller (BET) equation [26]. The total pore volume was estimated from the volume of N_2_ adsorbed at the relative pressure of 0.98 and converted it to the volume of N_2_ in the liquid state at a given temperature. Micropore volume was determined by applying the Dubinin–Radushkevich (DR) equation [27]. The mesopore volume was estimated by subtracting the micropore volume from the total pore volume. The pore size distributions of the derived activated carbons were determined from the N_2_ adsorption isotherms data by applying the Grand Canonical Monte Carlo (GCMC) simulation [25,28,29]. The average pore size (*D_av_*) was computed based on the equation 4*V_T_*/*S*, where *V_T_* is the total pore volume and *S* is the BET surface area, assuming the pores are cylinder. The contents of the oxygen functional groups on the activated carbon surfaces were additionally measured by applying the Boehm titration technique [30,31]. Details of the experimental and calculation procedures for the Boehm titration method are given elsewhere [25]. Moreover, pH at the point of zero charge (pH_PZC_) of the activated carbon was measured by the following procedure: 25 mL of 0.01 N NaCl solution was placed in an Erlenmeyer flask. The pH was adjusted to a value between 3 and 11 by using either HCl or NaOH. Then, 50 mg of the activated carbon sample was added to each of the sample solutions and shaken for 48 h at room temperature, and the final pH of each solution was measured. A plot of the final solution pH against the initial pH was made and the pH at which the plotted curve intersected the straight line of pH_initial_ = pH_final_ was taken as the point of zero charge (pH_PZC_) of the resulting carbon [32], as graphically shown in Figure S2 of the Supplementary Materials.

### 2.4. Methylene Blue Adsorption

The kinetics of MB adsorption was first performed to study the dye adsorption rate and to determine the equilibrium time of adsorption (the time at which the amount of dye adsorbed becomes constant) for the subsequent adsorption equilibrium experiments. The concentration of dye in the solution after adsorption was determined from a calibration curve based on the absorbance of a series of standard dye solutions of known concentrations. The absorbance was measured with a double beam ultraviolet-visible (UV-Vis) spectrophotometer (T80+ UV-Vis, PG Instrument Ltd., Leicestershire, UK) using the wavelength (λmax) of 665 nm. For the kinetics tests, 25 mL of the dye solution with an initial concentration of 200 mg/L was mixed with 0.02 g of activated carbon and shaken at a set temperature of 35 °C in a temperature-controlled water bath at 150 rpm. The solution sample was then collected over a time interval of up to 48 h and the collected sample was analyzed for the MB concentration using the measured UV absorbance and the prepared calibration curve. For the study of equilibrium adsorption isotherms, 25 mL of each dye solution with a concentration in the range of 50–500 mg/L was mixed with a fixed amount of activated carbon and shaken at a constant temperature of 35 °C for about 48 h (as determined by the kinetics study) to reach the equilibrium. Then, the final solution was collected for the analysis of the equilibrium dye concentration using the prepared calibration curve. The amount of activated carbon used for the adsorption experiments was carefully chosen to give the final equilibrium dye concentration of up to 300 mg/L. The equilibrium adsorption study was additionally carried out to study the effects of such process variables as adsorbent dosage, solution pH, and adsorption temperature. Table 1 summarizes the experimental conditions used for the study of MB adsorption by longan seed-activated carbons in the present study.

The adsorption capacity (*q_t_*) and the removal efficiency (*R_e_*) of MB by activated carbons was calculated using Equations (1) and (2), respectively:

(1) $$q_{t}=\frac{\left(C_{0}-C\right)V}{W}$$

(2) $$Re(\%)=\frac{\left(C_{0}-C\right)}{C_{0}}\times 100$$

where *q_t_* is the adsorption capacity of MB (mg/g) at time *t*, *R_e_* (*%*) is the removal efficiency of MB dye, *C*_0_ and *C* are the initial concentration and the concentration at time *t* of MB (mg/L), respectively, *V* is the volume of the solution (L), and *W* is the amount of longan seed-activated carbon employed (g). For adsorption at equilibrium, *C* = *C_e_* and *q_t_* = *q_e_*.

### 2.5. Adsorption Analysis

#### 2.5.1. Adsorption Kinetic Models

The kinetics of dye adsorption was analyzed using the two well-known rate equations, namely the pseudo-first-order kinetic model and the pseudo-second-order kinetic model [33,34], as shown in Equations (3) and (4), respectively:

(3) $$\frac{dq_{t}}{dt}=k_{1}\left(q_{e}-q_{t}\right)$$

(4) $$\frac{dq_{t}}{dt}=k_{2}{\left(q_{e}-q_{t}\right)}^{2}$$

Integrating the above equations gives Equations (5) and (6), respectively:

(5) $$q_{t}=q_{e}\left(1-e^{-k_{1}t}\right)$$

(6) $$q_{t}=\frac{q_{e}^{2}k_{2}t}{1+q_{e}k_{2}t}$$

where *q_e_* is the amount of dye adsorbed at equilibrium (mg/g carbon), *q_t_* is the amount adsorbed at time *t*, *k*_1_ (min^−1^) and *k*_2_ (g/mg·min) are the corresponding model rate constants.

The prediction of adsorption kinetics can also be achieved by employing a pore-diffusion model [35], in which the adsorption process is governed by the intraparticle diffusion of adsorbate molecules, neglecting the external or film mass transfer resistance. The final solution for the fractional uptake of adsorbate (*F*), assuming a spherical adsorbent, as shown in Equation (7):

(7) $$F(t)=\frac{q_{t}}{q_{e}}=1-\left(\frac{6}{\pi^{2}}\right)\sum_{n=1}^{\infty }\frac{1}{n^{2}}e^{-n^{2}\pi^{2}\tau }$$

where *F*(*t*) is the fractional uptake of the adsorbate at time *t*, *τ* is a non-dimensional time parameter defined as *τ* = *D_e_ t*/$R_{p}^{2}$, where *D_e_* is the average of effective pore diffusivity, and *R_p_* is the radius of the adsorbent particle (0.85 mm for this work). At the adsorption time close to equilibrium (*F* > 0.7), only the first term of the series in Equation (7) needs to be considered since the higher terms may be neglected [35]. Finally, this gives:

(8) $$F(t)=1-\left(\frac{6}{\pi^{2}}\right)e^{\frac{{}^{-\pi^{2}D_{e}t}}{R_{p}^{2}}}$$

The three kinetic model equations, Equations (5), (6) and (8), were used to fit the experimental kinetic data of MB adsorption to determine the kinetic model parameters that gave the best fitting results.

#### 2.5.2. Adsorption Isotherm Models

In designing an adsorption system, it is essential to use an accurate mathematical description of the adsorption isotherms. Several isotherm equations have been proposed for the adsorption of a wide variety of adsorbates from solutions by activated carbons [36]. The parameters of these isotherm equations indicate the nature of the surface heterogeneity and the affinity between the adsorbent and adsorbate at a fixed temperature and pH. In this study, the experimental isotherm data were fitted with the following isotherm models, namely the Langmuir isotherm [37], Freundlich isotherm [38], and Redlich–Peterson isotherm [39] equations to validate the prediction capability of the tested models, as shown in Equations (9)–(11), respectively:

(9) $$\mathrm{Langmuir}:\;q_{e}=\frac{q_{m}K_{L}C_{e}}{\left(1+K_{L}C_{e}\right)}$$

(10) $$\mathrm{Freundlich}:\;q_{e}=K_{F}C_{e}{}^{1/n_{F}}$$

(11) $$\mathrm{Redlich}–\mathrm{Peterson}:\;q_{e}=\frac{K_{R}C_{e}}{1+A_{R}C_{e}^{\beta }}$$

It should be noted that most adsorption models were originally developed for gas adsorption with certain assumptions. Therefore, when applying these models for liquid systems, they are used simply as empirical equations. The Langmuir equation was originally proposed for monolayer adsorption of a gas on a homogeneous flat surface (constant adsorption energy) and contains two model parameters, namely the monolayer capacity (*q_m_*, mg/g) and the Langmuir or affinity constant (*K_L_*, L/mg), which is a measure of how strong an adsorbate molecule is attracted onto an adsorbent surface. The two-parameter Freundlich equation was developed based on the assumption of monolayer adsorption on a patch-wise heterogeneous surface (distribution of adsorption energy). The Freundlich isotherm equation was originally developed as an empirical equation but it can also be derived based on thermodynamic consideration [40]. It also contains two model parameters, namely *K_F_* which corresponds to the binding capacity and *n_F_* which characterizes the surface heterogeneity. The larger the value of *n_F_*, the higher the heterogeneity of the surface and the more non-linearity of the isotherms. The Redlich–Peterson equation is a three-parameter empirical model that incorporates features of both the Langmuir and Freundlich equations. At low adsorbate concentrations, it follows a linear isotherm, and at high concentrations, its behavior approaches the Freundlich isotherm, which does not have a saturation limit. It can describe the adsorption process over a wide range of concentrations. *A_R_*, *β*, and *K_R_* are the model constants, where *β* is an exponent that lies between 0 and 1, and *K_R_* is the modified Langmuir constant (L/g). As a first approximation, it is generally assumed that *K_R_* is equal to *K_L_*.

The parameters of the isotherm and the kinetic models were determined by fitting the experimental data with the corresponding model equations using a non-linear regression analysis that minimizes the sum of squared estimate of errors (SSE) between the experimental and the computed values. The goodness-of-fit between the model and the experimental results was tested by the value of the regression coefficient, *R*^2^, and the normalized standard deviation (Δ*q*) which is defined by Equation (12) as follows,

(12) $$\Delta q(\%)=100\times \sqrt{\frac{{\sum \left[\left(q_{t,\exp}-q_{t,cal}\right)/q_{t,\exp}\right]}^{2}}{N-1}}$$

where the subscripts *exp* and *cal* denote the experimental and calculated values, respectively, and *N* is the number of data points. The higher the value of *R*^2^ and the lower the value of Δ*q*, the better the goodness-of-fit.

## 3. Results and Discussion

### 3.1. Characterization of the Prepared Activated Carbons

Table 2 shows the porous properties of the prepared microporous carbon series (A1–A3) and mesoporous carbon series (A4–A6). It should be noted that the activation temperature and time for each pair of A1 and A4, A2 and A5, and A3 and A6 are the same, that is 850 °C and 120 min, 850 °C and 180 min, and 850 °C and 240 min, respectively. Overall, the mesoporous-activated carbons produced by the OTA method had larger porous properties than those of microporous-activated carbons prepared by the two-step activation process under the same activation conditions (time and temperature). It is noticed that that the porous properties and the average pore size of activated carbons increased with the increase of activation time, but the percentage increase was more pronounced for the mesoporous carbons. Furthermore, when the activation time increased, the percentage of micropore volume decreased whereas that of the mesopore volume tended to increase. This indicates that the mesopores were created at the expense of newly formed micropores. The highest porous properties (*V_mic_* of 0.600 cm^3^/g, *V_mes_* of 0.474 cm^3^/g, *V_T_* of 1.074 cm^3^/g, and *S_BET_* of 1773 m^2^/g) were obtained with the activated carbon A6 produced by the three-cycle OTA method. Again, these results clearly show that the OTA method is more effective, as compared to the two-step activation, for the production of activated carbons with higher amounts of micropores and mesopores as well as a larger surface area.

Table 3 shows the distribution of pore sizes for the prepared activated carbons. The pore size distribution data showed the type of multimodal distribution, covering the pore sizes ranging from 0.65 to 4 nm. Most of the microporous carbons (A1–A3) were in the micropore size range (0.65–1.4 nm), followed by the upper mesopore size range of 3–4 nm. The volume of micropores (0.65–1.4 nm) decreased with the increase of activation time for carbons A1 to A3, while the opposite trend is observed for the supermicropores (1.4–2 nm). This observation indicates that supermicropores are responsible for the increase of surface area with increasing activation time. The upper mesopore size of 3–4 nm constituted most of the mesopore volume for A1 to A3 carbons, and this pore size range played a significant role in facilitating the diffusion of dye molecules in the activated carbon. The mesoporous carbons (A4–A6) also contained large proportions of micropores (0.65–1.4 nm) and most of the mesopores were concentrated in the lower size range of 2 to 3 nm. Intuitively, a large surface area of activated carbon should promote MB adsorption capacity, whereas a large volume of mesopores should enhance the kinetics of MB diffusion through internal pores.

Table 4 shows the point of zero charge (pH_PZC_) and the amounts of oxygen functional groups on the carbon surfaces of microporous- and mesoporous-activated carbons. The amounts of both acid and basic functional groups increased with increasing activation time for the microporous carbons (A1–A3) and with the increasing number of preparation cycles for the mesoporous carbons (A4–A6). The number of basic groups were higher than those of the acid groups for all carbon samples, possibly because CO_2_ was used as the activating agent rather than water vapor (steam) [41]. The increasing amounts of basic groups for samples A1 to A3 and samples A4 to A6 indicate the increasing degree of basicity of the carbon surface, and this coincides with the increasing values of pH_PZC_ of the activated carbon, as expected. It is further noted that the mesoporous carbons had fewer acid groups and higher basic groups, as compared to microporous carbons. The lower number of surface acid groups of mesoporous carbons could result from the high-temperature treatment of the oxidized carbon as part of the OTA method that may have removed most of the acid functional groups prior to the following activation step.

**Figure 1:**
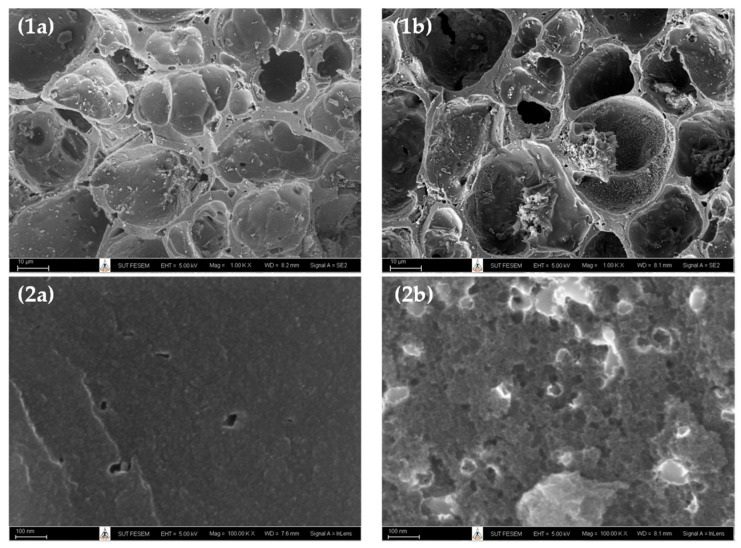
SEM micrographs of (**a**) the microporous-activated carbon of A2 (A850–180), and (**b**) the mesoporous-activated carbon of A5 (A850–60–2), with magnification of 1K for (**1a**–**1b**) and 100 K for (**2a**–**2b**).

**Figure 2:**
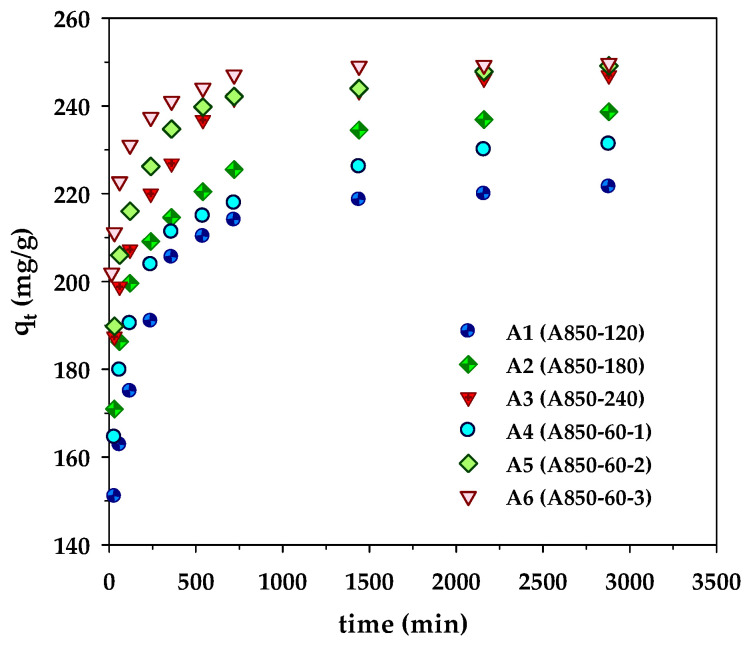
Kinetics of methylene blue adsorption on the prepared microporous- and mesoporous- activated carbons from longan fruit seed.

Figure 1 shows the surface images obtained from a field emission scanning electron microscope (FE-SEM, Carl Zeiss AURIGA^®^, Oberkochen, Germany) of microporous-activated carbon prepared by the two-step activation method (sample A2 or A850–180) and the mesoporous-activated carbon prepared by the OTA method (sample A5 or A850–60–2). Figure 1a,b and Figure 2a,b present the images at low and high magnifications, respectively. It can be seen that there was a tendency for an increase in the average pore size and the number of pores for the mesoporous carbon (A5), as compared with the microporous carbon (A2). These results are in line with those of the average pore size and pore volume results, as presented in Table 2.

### 3.2. Adsorption Kinetics and Model Testing

The kinetic results of MB adsorption by the microporous (A1–A3) and mesoporous carbons (A4–A6) are displayed in Figure 2. The kinetic curves showed a rapid increase of the amounts adsorbed over the first 500 min, followed by a slow increase before attaining an equilibrium at approximately 2000 min. However, the amount of MB adsorbed by the sample A850–60–3 (A6) approached the equilibrium sooner at 1500 min, obviously resulting from its largest average pore diameter (2.42 nm) that allows a faster diffusion rate of dye molecules to the adsorption sites. The amount of dye adsorbed appeared to be higher for the adsorbent with a higher surface area (see Table 2). This indicates that the adsorption sites are distributed more or less uniformly on the carbon surfaces, irrespective of the carbon surface area. In other words, the density of adsorption sites (number per unit area) is approximately constant. The best-fitted model parameters are listed in Table 5. From the values of *R*^2^ and Δ*q*, it is clear that the experimental kinetic data of MB adsorption by longan seed-activated carbons are best described by the pseudo-second-order model, followed by the pore-diffusion model and the pseudo-first-order model, respectively. The success of the pseudo-second-order model in describing the kinetics of MB adsorption was also reported for activated carbons prepared from various precursors such as abelmoschus esculentus seeds [42], black cumin seeds [43], and lychee seed [44].

Results from Table 5 indicate that the rate constants *k*_1_ and *k*_2_, as well as the effective pore diffusivity (*D_e_*), all increased with an increase in the average pore diameter of samples A1 to A3 and A4 to A6. This was anticipated since the larger pore sizes would be able to accommodate the rapid diffusion of relatively large dye molecules. It should also be noted that the pore diffusivities of mesoporous-activated carbons in the range of 4.67 × 10^−7^–19.92 × 10^−7^ cm^2^/s were larger than those of the microporous carbons (4.66 × 10^−7^–6.01 × 10^−7^ cm^2^/s) by almost an order of magnitude which should result from higher amounts of mesopores of the former that lower the mass transfer resistance for the transport of dye molecules through the pore network (see Table 2). The molecular diffusivity of methylene blue in water at 35 °C, as estimated by the Wilke–Chang equation [45], is about 5.57 × 10^−6^ cm^2^/s, which is about ten times and six times larger than the average pore diffusivities of the microporous carbon and mesoporous carbon, respectively. This suggests that the process of MB adsorption is controlled by the intra-particle diffusion of MB molecules, since the adsorption rate is generally much faster than the internal mass transport rate of the adsorbate molecules [46].

Closer examination of pore size distribution data in Table 3 reveals that the largest pore diffusivity of 19.92 × 10^−7^ cm^2^/s for sample A6 prepared by the three-cycle OTA method is associated with the large proportions of supermicropores (36.6% by volume) and mesopores of 2–3 nm (30.7%). Therefore, supermicropores (1.4–2 nm) and small mesopores (2–3 nm) would play an important part in controlling the adsorption kinetics of methylene blue adsorption by activated carbon with a high surface area and a high mesopore volume. The dominant transport mechanism of methylene blue molecules in the supermicropores is possibly the result of Knudsen diffusion, since the molecular size of methylene blue, 1.447 nm [47], is comparable to the pore size. The diffusion of MB in the small mesopores (2–3 nm) which have a pore size twice that of the molecular size of methylene blue could involve both the Knudsen diffusion and molecular diffusion. It should be realized that the optimum pore size distribution of activated carbon that leads to rapid kinetic behavior does not necessarily provide the maximum adsorption capacity because the maximum adsorption will depend on the available surface area and the number of adsorption sites.

**Figure 3:**
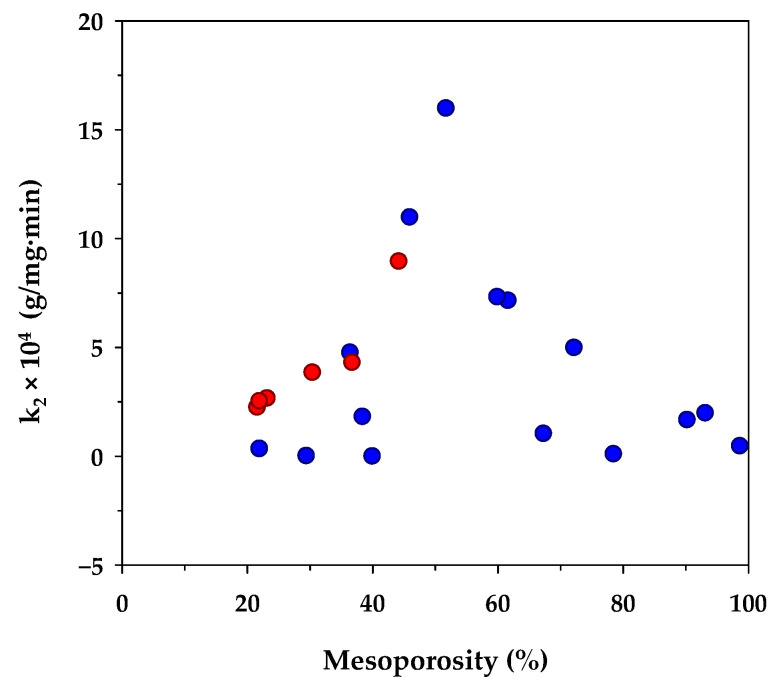
Effect of percent mesoporosity in activated carbons on the rate constant (*k*_2_) of the pseudo-second-order kinetic model. The red circle symbols represent the results from the present study and the blue circle symbols are those from other investigators (see references in Table 6).

Table 6 lists the value of *k*_2_ of the pseudo-second-order kinetic model derived from the present and previous studies of methylene blue adsorption by activated carbons prepared from different biomass precursors and the effect of the percentage of mesopore volume on *k*_2_ is shown in Figure 3. Although there are differences in the porous properties of activated carbons prepared from various sources of raw materials, as well as the adsorption conditions used, there appears an optimum percentage of mesopore volume of around 50% that yields a maximum rate constant *k*_2_. From these limited data, it could be deduced that the kinetics of MB diffusion in the porous structure of activated carbon is determined primarily by the relative proportion of micropores and mesopores. A smaller amount of mesopores tends to lower the diffusion rate of methylene blue due to an increase in the mass transfer resistance offered by the larger amounts of micropores. Since the transport of an adsorbate through the pore network and the adsorption on the carbon surface occur in series, the lower adsorption rate caused by the decrease of the surface area of larger mesopore volume would therefore lower the diffusive flux of the dye molecules inside the pores, and hence giving a relatively low value for *k*_2_.

### 3.3. Adsorption Isotherms and Model Testing

**Figure 4:**
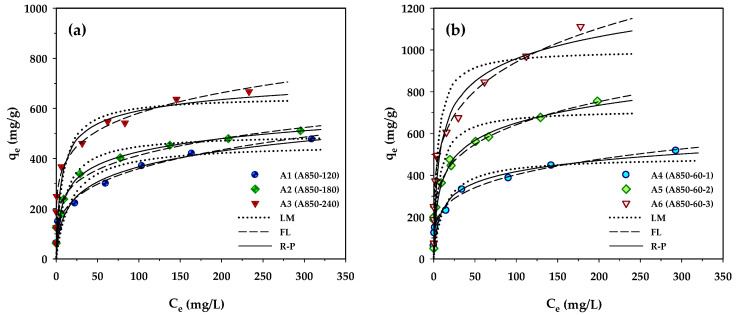
Adsorption isotherms at 35 °C of MB dye on longan seed-activated carbons prepared by (**a**) the two-step activation method, and (**b**) the OTA method.

Adsorption isotherms provide essential information for adsorption capacity and adsorption behavior of porous adsorbents. In the present study, the measured isotherm data for MB adsorption by the prepared activated carbons were tested with the Langmuir, Freundlich, and Redlich–Peterson equations, and the best-fitted model parameters achieved by applying regression analysis are listed in Table 7. Figure 4 compares the measured and the model-predicted isotherms for MB adsorption by the longan seed-activated carbons. Based on visual observation of Figure 4 and the values of *R*^2^ and Δ*q*, the Langmuir equation gave the least prediction capability of MB isotherms for all carbons. It is interesting to note that the Redlich–Peterson model can best describe the isotherms of microporous carbons prepared by the two-step activation (A1–A3), while the Freundlich equation is most appropriate for predicting the MB adsorption by the mesoporous carbons produced by the OTA method (A4–A6). The better description of MB adsorption isotherms by the Redlich–Peterson equation for the microporous carbons is possibly due to their higher percentage of micropore volume (see Table 2), thus showing a sharper linear isotherm behavior at low concentrations by micropore-filling adsorption. The ability of the Freundlich equation in better predicting the isotherm behavior of the mesoporous carbons may arise from the higher degree of heterogeneity of the carbon surfaces.

**Figure 5:**
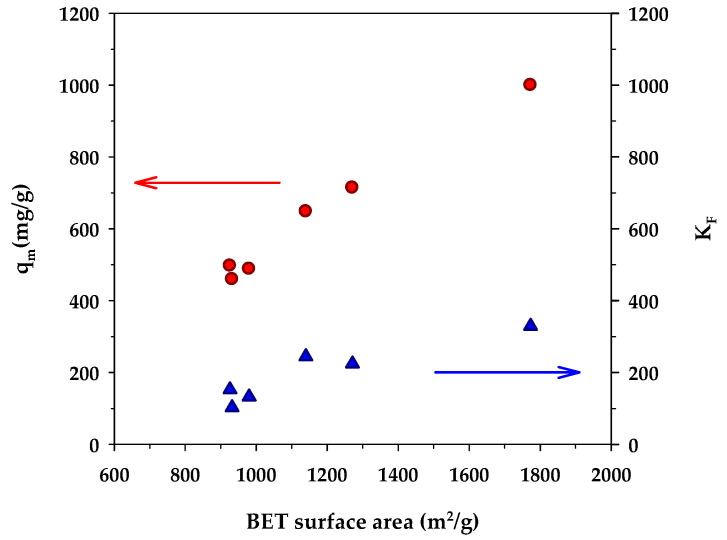
Effect of BET surface area on monolayer capacity (*q_m_*) of the Langmuir isotherm equation and *K_F_* of the Freundlich equation.

**Figure 6:**
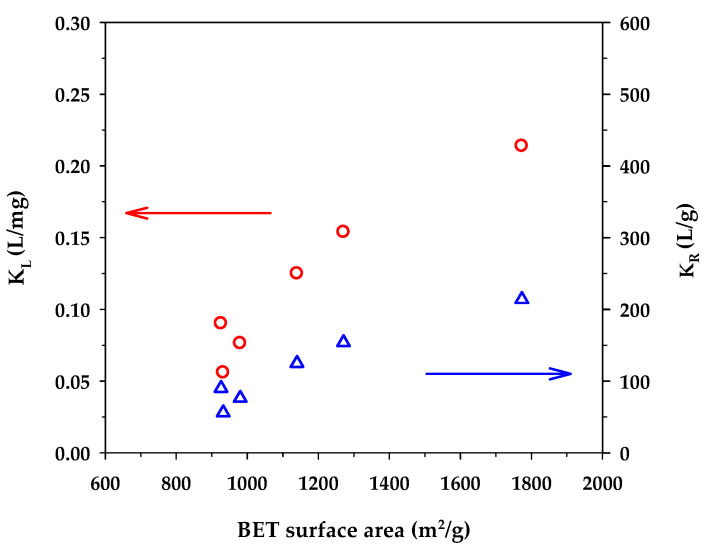
Effect of BET surface area on *K_L_* of the Langmuir equation and *K_R_* of the Redlich–Peterson equation.

Figure 5 shows the dependence of the binding capacity parameters, that is, the monolayer capacity (*q_m_*) of the Langmuir model and *K_F_* of the Freundlich model, on the surface area of activated carbons. Both parameters increased with an increase in the specific surface area. This is understandable since a greater surface area could provide a larger number of adsorption sites for the methylene blue dye. From Table 7, the surface heterogeneity parameter of the Freundlich equation (*n_F_*) appeared to be almost insensitive to the change in surface area, which suggests that the distribution of adsorption energy as a function of adsorption sites is to a certain degree almost independent of the developed surface area of activated carbons. Figure 6 shows the effect of the surface area of activated carbons on the affinity coefficients, *K_L_* and *K_R_*. Both parameters increased with the increase of surface area in the same fashion as that observed with the binding capacity parameters. The development of surface area due to the removal of carbon atoms from the graphene layer by gasification may create surface defects with stronger interaction forces, thus providing stronger adsorbent–adsorbate affinity. Furthermore, it can be seen from Table 7 that under the same activation time and temperature, the monolayer capacity (*q_m_*) of the Langmuir equation for the mesoporous carbons is higher than that of the microporous-activated carbons. For comparison, the percentage increase of *q_m_* from A1 to A4, A2 to A5, and A3 to A6 are 6.3%, 43%, and 54%, respectively. The highest value of the monolayer capacity of 1000 mg/g for MB adsorption was observed with the mesoporous-activated carbon prepared by the three-cycle OTA method (sample A6).

**Figure 7:**
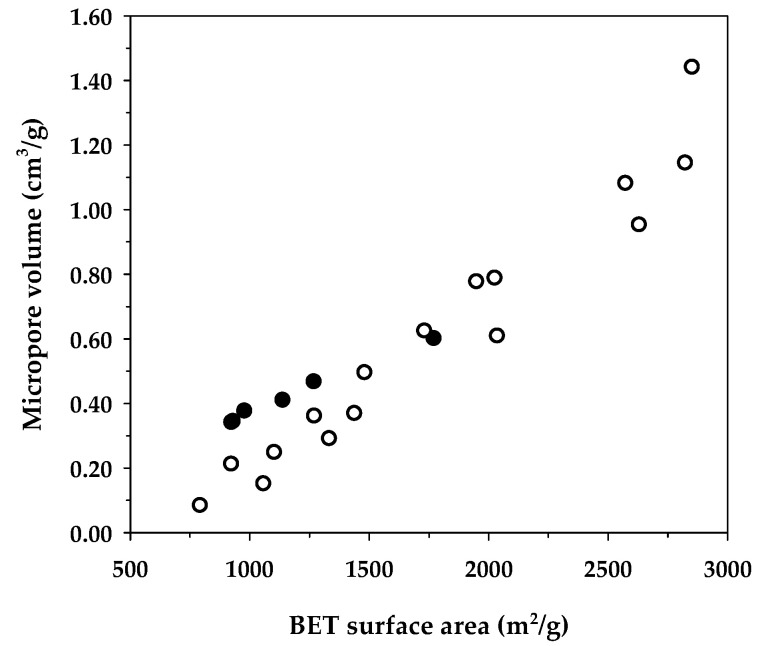
Correlation between the specific surface area and the maximum adsorption capacity of methylene blue from the data in Table 8. The black circles represent the activated carbons in the present study, whereas the white circles belong to previous investigations.

**Figure 8:**
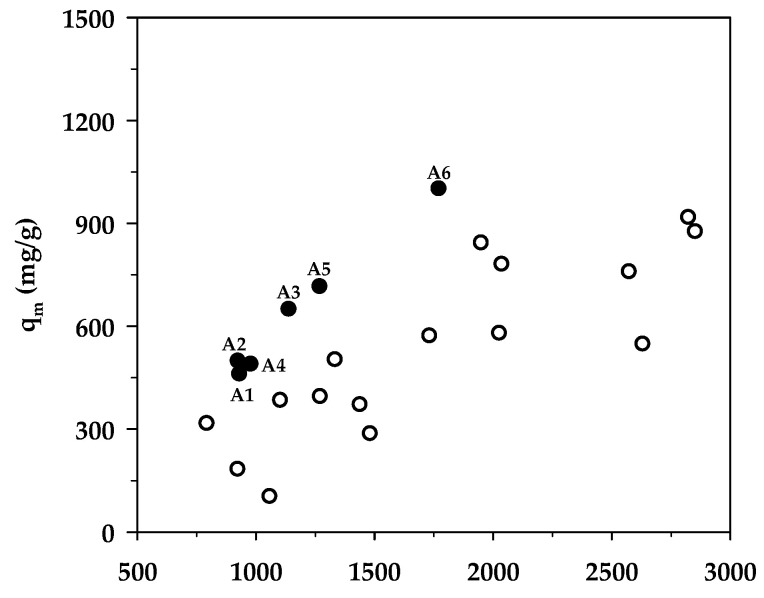
Effect of BET surface area on the maximum MB adsorption capacity. The black circles represent results from the present study (A1–A6), whereas the white circles represent those from previous works.

Table 8 compares the maximum adsorption capacity of MB adsorption by activated carbons from the present and previous investigations, together with the porous properties of the relevant adsorbents. It was observed that the percentage of micropore volume and mesopore volume varied from 13.2–90.0% and 10.0–86.8%, respectively, depending on the raw materials and the preparation conditions employed. As shown in Figure 7, the BET surface area increased almost linearly with an increase in micropore volume, emphasizing that the surface area of activated carbons is determined primarily by the amount of micropores. Figure 8 shows a plot of the maximum capacity of MB adsorption as a function of the surface area of activated carbons from the present and previous studies. The adsorption capacity increased with the increase of surface area as expected, since an adsorption process is a surface phenomenon of adsorbent–adsorbate interactions. It is seen that all data points from the present study were above those of the previous results, notably with the A6 carbon that gave the largest value of the adsorption capacity of 1000 mg/g. The relatively larger adsorption capacity of mesoporous carbons prepared by the OTA method (A4–A6) clearly indicates that activated carbons produced by the OTA method contain a larger number of adsorption sites. Additionally, the ratio of *q_m_/S_BET_* in Table 8 for mesoporous carbon A6 from this study remarkably showed that its ratio of 0.564 was about twofold higher than the average value of 0.296 for activated carbons reported by previous studies.

### 3.4. Effect of Process Variables on Methylene Blue Adsorption

#### 3.4.1. Effect of Adsorbent Dosage

**Figure 9:**
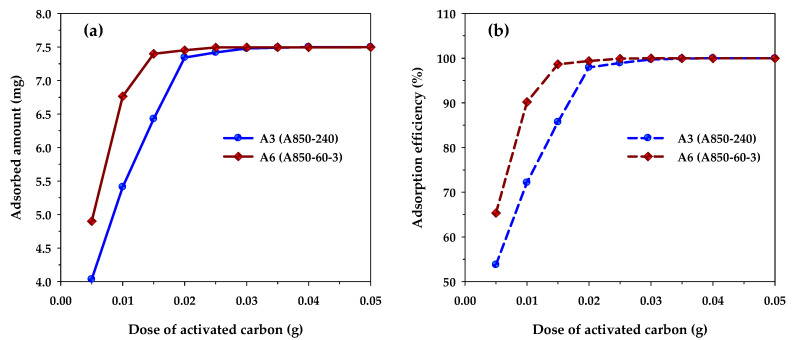
The effect of carbon dosage on (**a**) MB adsorption capacity and (**b**) MB removal efficiency for microporous (A3) and mesoporous (A6) activated carbons (*C*_0_ = 300 mg/L, *V* = 25 mL, and *T* = 35 °C).

Figure 9 shows the effects of carbon dosage on the adsorption capacity and removal efficiency of methylene blue when the performances of microporous carbon A3 and mesoporous carbon A6 are compared with both carbons being produced at the same activation time and temperature at 240 min and 850 °C, respectively. The adsorption conditions used were initial MB concentration (*C*_0_) of 300 mg/L, solution volume (*V*) of 25 mL, and adsorption temperature (*T*) of 35 °C. Figure 9a shows that the amount of MB adsorbed in mg increased with an increase of carbon dosage and became constant when the dosage reached a certain maximum value. Clearly, the increase in the amount adsorbed is the result of an increase in the number of adsorption sites provided by the increase in the amount of the adsorbent. Therefore, when the number of adsorption sites is in excess of those required for adsorption, the amount of MB adsorbed will reach a maximum and further addition of the adsorbent will exert no effect on the amount of adsorption. It can be seen from Figure 9a that the maximum carbon dosage for the mesoporous carbon and microporous carbon were 0.02 and 0.03 g, respectively. When multiplying the dosage with the corresponding BET surface area, the maximum area required for the maximum amount adsorbed were 34.2 m^2^ (0.03 g × 1140 m^2^/g) and 35.5 m^2^ (0.02 g × 1773 m^2^/g) for the microporous and mesoporous carbons, respectively, which are approximately the same. This clearly indicates that the porous property of surface area is important for determining the adsorption capacity of methylene blue dye, since the larger the surface area of the adsorbent, the higher the amount of the dye being adsorbed. It should be noted that before reaching the maximum adsorption, the amount of MB adsorbed was higher for the mesoporous carbon compared to that of the microporous carbon. Obviously, this is the result of the larger surface area of the mesoporous carbon that can provide a larger number of adsorption sites. Figure 9b shows that the removal efficiency of methylene blue, calculated according to Equation (2), increased with the increase in the amount of adsorbent used. The trend of the curves was the same as that for the amount of MB adsorbed as shown in Figure 9a. The maximum removal efficiency of MB at the maximum carbon dosage was 99.71 and 99.90% for the microporous and mesoporous carbons, respectively. This indicates that activated carbon is suitable for the effective removal of an organic dye such as methylene blue.

#### 3.4.2. Effect of Initial pH

**Figure 10:**
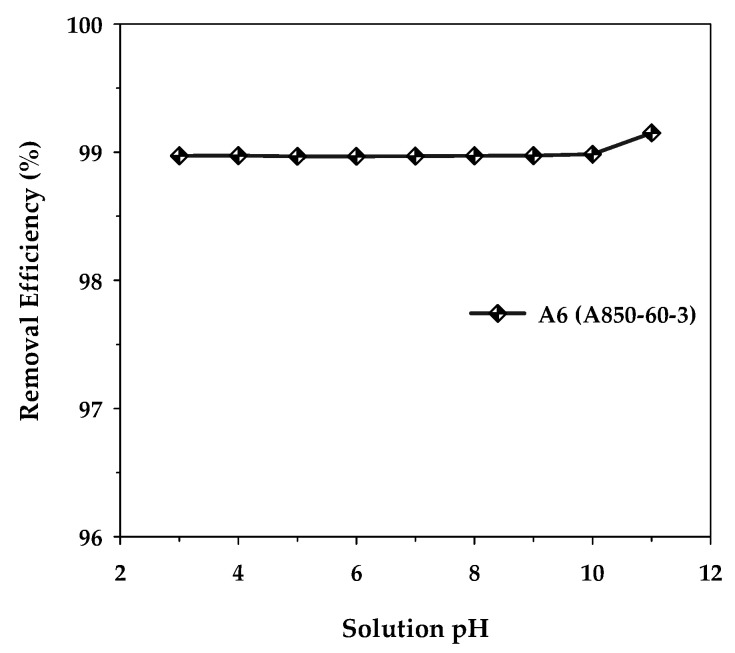
Effect of the initial solution pH on the removal efficiency of MB by the mesoporous-activated carbon (A6), with *C*_0_ = 400 mg/L, carbon dosage = 0.8 g/L, and *T* = 35 °C.

The effect of the initial pH of the MB solution on the removal efficiency of methylene blue dye was investigated using activated carbon with a maximum surface area, mesoporous carbon A6, by varying the initial pH in the range of 3 to 11 under fixed conditions of adsorbent dosage of 0.8 g/L, initial dye concentration of 400 mg/L, adsorption time of 48 h, and the temperature of 35 °C. Figure 10 shows the results obtained. It can be observed that the adsorption capacity remained substantially constant over the pH range of 3–10. However, when the solution pH increased from the value of 10 to 11, the removal efficiency increased slightly from 98.98% to 99.15%, corresponding to the amount of MB adsorbed from 494.92 to 495.75 mg/g. This increase could arise as a result of the adsorbent surface becoming more negatively charged at a pH above 10, since the solution pH is higher than the experimentally determined pH_PZC_ of 9.94 for the activated carbon. This would enhance the strong electrostatic attraction between the cationic MB dye and the negatively charged carbon surface. Therefore, the slight effect of the solution pH on the adsorption efficiency of methylene blue seems to suggest that the electrostatic attraction alone may not be the primary mechanism for the adsorption of methylene blue onto the activated carbon. The other types of underlying interactions between methylene blue and the carbon surface will be briefly outlined in the next section.

#### 3.4.3. Effect of Temperature and Adsorption Thermodynamics Study

**Figure 11:**
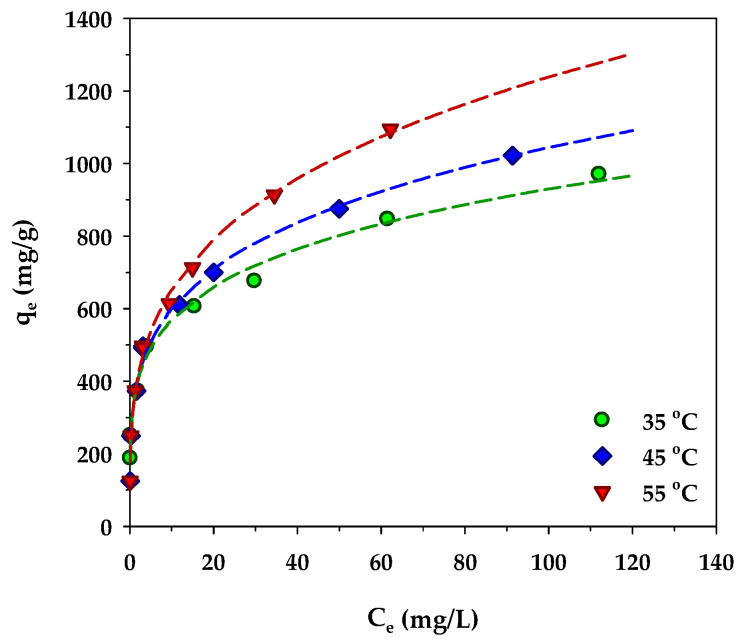
Effect of temperature on isotherms of MB adsorption by the mesoporous-activated carbon (A6).

The variation of MB adsorption isotherms of the mesoporous-activated carbon, A6, as a function of temperature is displayed in Figure 11. Over the temperature range from 35 to 55 °C, the amount of MB adsorbed increased with the increase in the adsorption temperature. The role of the temperature in adsorption is twofold, that is, it can affect the rate of adsorbate diffusion through the internal pores of an adsorbent and the adsorption ability of the adsorbate on the adsorbent surface via some kind of interaction forces [72]. Three dominant interactions between methylene blue molecules and the surface of activated carbon have been reported [73]. They are (i) π-π interactions between the aromatic rings of MB and the graphene sheet of activated carbon, (ii) hydrogen bonding between the hydrogen in the hydroxyl group on the carbon surface and the nitrogen in the MB structure, and (iii) electrostatic interactions between electron-deficiency nitrogen (N^+^) in the MB structure and electron-rich oxygen (O^−^) of the surface functional groups on activated carbon. Since it is known that the magnitude of these interaction forces decreases with an increase in temperature [74,75], thus the increasing amount of MB adsorbed with the increase in temperature must be related to its effect on the transport of the methylene molecules in the carbon pore structure. It is likely that the increase of temperature would increase the diffusion rate of methylene blue molecules to the adsorption sites that would increase the likelihood of an adsorbent–adsorbate interaction, hence the increasing amount of methylene blue adsorbed. A similar temperature effect on the amount of MB adsorbed was also observed by a number of investigators working on different adsorbents, for example, tannin [76], rejected tea [77], magnetic graphene-CNTs [78], and N and S co-doped porous carbon spheres [79].

**Figure 12:**
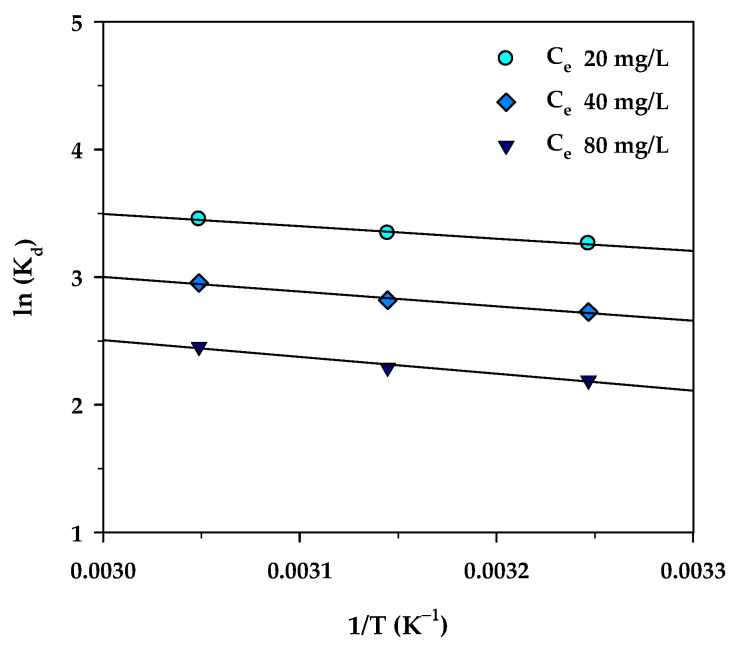
The plot of *ln*(*K_d_*) versus 1/*T* for MB adsorption onto mesoporous-activated carbon (A6).

In order to gain a fuller understanding of the dye adsorption process, the various thermodynamic parameters including the values of enthalpy change (Δ*H*°), Gibbs free energy change (Δ*G*°), and entropy change (Δ*S*°) were estimated from the isotherm data at various temperatures by using the following equations [80]:

(13) $$\ln K_{d}=\frac{\Delta S^{{}^{\circ}}}{R}-\frac{\Delta H^{{}^{\circ}}}{RT}$$

(14) $$\Delta G^{{}^{\circ}}=-RT\ln K_{d}$$

where *R* is the universal gas constant (8.314 J/mol∙K), *T* (K) is the absolute solution temperature, and *K_d_* is the distribution coefficient for the adsorption, which is the ratio of the amount of MB in the adsorbed phase and that in the aqueous phase, and which can be computed from the relation, *K_d_* = (*q_e_/C_e_*)·(*W/V*). Figure 12 shows plots of *lnKd* versus *1/T* for three different values of *C_e_* (20, 40, and 80 mg/L). According to Equation (13), the values of Δ*H°* and Δ*S°* were then determined from the slope and intercept of the straight-line graph. Equation (14) was used for the calculation of the Gibbs free energy change (Δ*G°*) at various temperatures.

The estimated thermodynamic parameters for MB adsorption by the mesoporous carbon (A6) are summarized in Table 9. The values of the three thermodynamic parameters changed over a narrow range with respect to the change in the equilibrium concentration of the MB solution. The positive values of Δ*H°* indicate that the adsorption of methylene blue and activated carbon in this work is an endothermic process, in accordance with the increase of MB adsorbed with increasing temperature. In addition, the relatively low enthalpy change (Δ*H°*) in the range of 8.03–10.98 kJ/mol indicates that the adsorption of MB on the carbon surface occurs by the physisorption process through intermolecular forces [81]. Furthermore, the positive adsorption entropy (Δ*S°*) indicates the affinity of the adsorbate towards the adsorbent which reflects an increase in randomness at the solid/solution interface during the adsorption process. The negative free energy change (Δ*G°* < 0) indicates a favorable process of adsorption and the spontaneous nature of methylene blue uptake over the temperature range studied.

## 4. Conclusions

Microporous- and mesoporous-activated carbons were produced from longan fruit seeds by the two-step activation and the OTA method, respectively, using carbon dioxide as the activating agent. Micropores of size range 0.65–1.4 nm and the upper mesopore size of 3–4 nm constituted most of the pores in the microporous-activated carbons, whereas the mesoporous carbons contained most of the pores in the micropore size of 0.65–1.4 nm and the smaller mesopore size of 2–3 nm. The maximum porous properties of activated carbon, including surface area (1773 m^2^/g), micropore volume (0.600 cm^3^/g), mesopore volume (0.474 cm^3^/g), and the average pore size (2.42 nm) were derived from the mesoporous carbon produced by the three-cycle OTA method. During the transient adsorption of MB from the solution, the amount of MB adsorbed increased proportionally with the increase in time and the carbon surface area. The pseudo-second-order kinetic model was found to provide the best description for the kinetics of MB adsorption, followed by the pore-diffusion model and the pseudo-first-order model, respectively. The rate constants *k*_1_ of the pseudo-first-order model, *k*_2_ of the pseudo-second-order model, and the effective pore diffusivity (*D_e_*) of the pore-diffusion model all increased with the increase in the average pore size, which can be explained by the reduction of mass transfer resistance with increasing pore size. The average pore diffusivity of the mesoporous carbon was found to have a value of 11.8 × 10^−7^ cm^2^/s which was about an order of magnitude larger than that of the microporous carbon.

The adsorption isotherms of methylene blue by the microporous and mesoporous carbons are best described by the Redlich–Peterson and Freundlich equations, respectively. The maximum MB adsorption capacity of 1000 mg/g was achieved with the highest surface area of the mesoporous carbon. The initial pH of the MB solution had virtually no effect on the adsorption capacity or the removal efficiency of methylene blue by the activated carbons tested. An increase in the adsorption temperature over the range from 35 to 55 °C gave rise to an increase in the amount of MB adsorbed, which was presumably caused by the increase in the diffusion rate of methylene blue molecules to the adsorption sites that provided an increase in the frequency of adsorbent–adsorbate interactions, hence leading to successful adsorption. A thermodynamic analysis of the adsorption revealed that the MB adsorption by longan seed-based activated carbon is an endothermic, favorable, and physically adsorbed process. In summary, the high surface area of mesoporous-activated carbon prepared from longan seed biomass in this study has been proved to be highly effective for the adsorption of methylene blue from aqueous solution from the perspective of rapid kinetics and high adsorption equilibrium capacity.

## Figures and Tables

**Table 1:** Experimental conditions for the study of batch MB adsorption by longan seed-based activated carbons.

Studied Variables	Time	Initial Concentration	Carbon Dosage	Temperature	pH
	(h)	(mg/L)	(g)	(°C)	
Time (kinetics)	0.5–48	200	0.020	35	4.5
Equilibrium conc.	48	50–500	0.010–0.020	35	4.5
Adsorbent dosage	48	300	0.005–0.050	35	4.5
Solution pH	48	400	0.020	35	3–11
Temperature	48	50–500	0.010–0.020	35–55	4.5

**Table 2:** Porous properties of prepared microporous- and mesoporous-activated carbons.

Sample	Sample	*D_av_*	*V_Mic_*	*V_Mes_*	*V_T_*	*S_BET_*
Code	Name	(nm)	(cm^3^/g) (%)	(cm^3^/g) (%)	(cm^3^/g)	(m^2^/g)
A1	A850–120	1.88	0.343 (78.5)	0.094 (21.5)	0.437	932
A2	A850–180	1.91	0.340 (76.9)	0.102 (23.1)	0.442	926
A3	A850–240	2.06	0.409 (69.7)	0.178 (30.3)	0.587	1140
A4	A850–60–1	1.96	0.375 (78.1)	0.105 (21.9)	0.480	980
A5	A850–60–2	2.32	0.466 (63.3)	0.270 (36.7)	0.736	1271
A6	A850–60–3	2.42	0.600 (55.9)	0.474 (44.1)	1.074	1773

**Table 3:** Pore size distribution of prepared activated carbons.

Sample	*V_T_*	Pore Volume for Pore Width (cm^3^/g)			
Code	(cm^3^/g)	0.65–1.4 nm	1.4–2 nm	2–3 nm	3–4 nm
A1	0.437	0.306 (70.0%)	0.037 (8.5%)	0.006 (1.4%)	0.088 (20.1%)
A2	0.442	0.291 (65.8%)	0.049 (11.1%)	0.002 (0.5%)	0.100 (22.6%)
A3	0.587	0.307 (52.3%)	0.102 (17.4%)	0.012 (2.0%)	0.166 (28.3%)
A4	0.480	0.309 (64.4%)	0.066 (13.7%)	0.046 (9.6%)	0.059 (12.3%)
A5	0.736	0.339 (46.1%)	0.127 (17.2%)	0.176 (23.9%)	0.094 (12.8%)
A6	1.074	0.207 (19.3%)	0.393 (36.6%)	0.330 (30.7%)	0.144 (13.4%)

**Table 4:** Point of zero charge and surface functional groups in activated carbons.

Sample	pH_PZC_	Acidic Groups	Basic Groups	Total
Code		(mmol/g)	(mmol/g)	(mmol/g)
A1	8.77	0.243 (20.5%)	0.941 (79.5%)	1.184
A2	8.81	0.334 (22.4%)	1.160 (77.6%)	1.494
A3	9.48	0.400 (23.1%)	1.329 (76.9%)	1.729
A4	9.36	0.152 (10.5%)	1.295 (89.5%)	1.447
A5	9.54	0.218 (14.1%)	1.331 (85.9%)	1.549
A6	9.94	0.251 (14.4%)	1.491 (85.6%)	1.742

**Table 5:** Kinetic parameters of various adsorption kinetic models for MB adsorption by longan seed-activated carbons.

Code	Pseudo-First Order				Pseudo-Second Order				Pore-Diffusion			
	*q_e_*	*k*_1_	*R* ^2^	Δ*q*	*q_e_*	*k*_2_ × 10^4^	*R* ^2^	Δ*q*	*q_e_*	*D_e_* × 10^7^	*R* ^2^	Δ*q*
	(mg/g)	(min^−1^)		(%)	(mg/g)	(g/mg·min)		(%)	(mg/g)	(cm^2^/s)		(%)
A1	206.98	0.034	0.914	8.94	222.22	2.265	1.000	4.39	218.52	4.657	0.987	5.38
A2	221.63	0.042	0.912	6.81	238.10	2.669	1.000	4.40	235.98	4.667	0.957	5.14
A3	232.43	0.047	0.819	6.81	240.45	3.850	1.000	3.57	244.76	6.014	0.990	4.26
A4	215.08	0.041	0.947	7.00	232.56	2.548	1.000	4.06	226.94	4.668	0.984	4.02
A5	236.40	0.048	0.895	5.39	243.15	4.312	1.000	2.51	241.39	10.762	0.989	3.85
A6	239.91	0.110	0.805	5.09	246.60	8.962	1.000	1.82	248.30	19.920	0.974	3.74

**Table 6:** Comparison of the kinetic parameter (*k*_2_) of the pseudo-second-order model for various types of activated carbons.

Biomass Precursors	Activating	*C*_0_	*T*	*S_BET_*	*V_mic_*	*V_mes_*	*V_T_*	*k*_2_ × 10^4^	Ref.
	Agent	(mg/L)	(°C)	(m^2^/g)	(cm^3^/g) (%)	(cm^3^/g) (%)	(cm^3^/g)	(g/mg·min)	
Mangosteen peel	ZnCl_2_	100	25	890	0.010 (1.4)	0.701 (98.6)	0.711	0.480	[48]
Coconut leaves	H_3_PO_4_	200	30	982	0.095 (6.9)	1.276 (93.1)	1.371	2.000	[49]
Jerusalem artichoke	ZnCl_2_	200	30	1632	0.120 (9.8)	1.100 (90.2)	1.220	1.678	[50]
Vetiver roots (P1.5)	H_3_PO_4_	-	25	1004	0.220 (21.6)	0.800 (78.4)	1.020	0.110	[51]
Vetiver roots (P1.0)	H_3_PO_4_	-	25	1272	0.390 (32.8)	0.800 (67.2)	1.190	1.050	[51]
Rattan stalks	NaOH	250	30	1135	0.170 (27.9)	0.440 (72.1)	0.610	5.000	[52]
Chitosan flakes	NaOH	200	30	318	0.098 (38.4)	0.157 (61.6)	0.255	7.160	[53]
Orange peel	K_2_CO_3_	200	30	1104	0.247 (40.2)	0.368 (59.8)	0.615	7.333	[54]
*Posidonia oceanica*	ZnCl_2_	750	25	1483	0.494 (48.3)	0.528 (51.7)	1.022	16.000	[55]
*Dipterocarpus alatus*	ZnCl_2_	6	-	843	0.256 (54.1)	0.217 (45.9)	0.473	10.990	[56]
Waste tea	CH_3_CO_2_K	150	30	854	0.310 (60.1)	0.206 (39.9)	0.516	0.010	[57]
Coconut shell	NaOH	200	30	876	0.272 (61.7)	0.169 (38.3)	0.441	1.833	[58]
Eucalyptus sawdust	FeCl_3_	150	35	645	0.280 (63.6)	0.160 (36.4)	0.440	4.770	[59]
*Thevetia peruviana*	KOH	100	40	588	0.342 (70.7)	0.142 (29.3)	0.484	0.031	[60]
Durian shell	KOH	200	40	992	0.368 (78.1)	0.103 (21.9)	0.471	0.357	[61]
Longan seed (A6)	CO_2_	200	35	1773	0.600 (55.9)	0.474 (44.1)	1.074	8.962	–

**Table 7:** Parameters of adsorption isotherm equations for MB adsorption at 35 °C by the prepared activated carbons.

Code	Langmuir				Freundlich				Redlich–Peterson				
	*q_m_*	*K_L_*	*R* ^2^	Δ*q*	*n_F_*	*K_F_*	*R* ^2^	Δ*q*	*K_R_*	*β*	*A_R_*	*R* ^2^	Δ*q*
	(mg/g)	(L/mg)		(%)				(%)	(L/g)				(%)
A1	459.35	0.056	0.984	24.39	3.67	102.6	0.992	8.04	56.0	0.81	0.34	0.999	5.87
A2	497.12	0.090	0.995	5.45	4.61	152.2	0.957	12.46	90.2	0.86	0.38	0.998	4.29
A3	648.12	0.125	0.989	12.60	5.31	244.2	0.980	11.75	125.0	0.93	0.28	0.992	5.74
A4	488.29	0.076	0.987	22.80	4.13	132.1	0.996	4.69	76.3	0.86	0.32	0.970	7.54
A5	714.29	0.154	0.986	15.42	4.38	224.2	0.996	4.88	153.8	0.85	0.45	0.989	9.40
A6	1000.00	0.214	0.986	18.47	4.38	328.9	0.991	6.82	214.0	0.88	0.37	0.981	12.39

**Table 8:** The maximum adsorption capacity of methylene blue from solutions (*q_m_*) by activated carbons prepared from various materials and activation methods.

Raw Materials	Activating	*S_BET_*	*V_mic_*	*V_mes_*	*V_T_*	*D_av_*	*q_m_*	*q_m_/S_BET_*	Ref.
	Agent	(m^2^/g)	(cm^3^/g) (%)	(cm^3^/g) (%)	(cm^3^/g)	(nm)	(mg/g)	(mg/m^2^)	
Flamboyant pods	NaOH	2854	1.44 (90.0)	0.160 (10.0)	1.600	2.24	874.7	0.306	[62]
Coconut shell	NaOH	2825	1.143 (76.3)	0.355 (23.7)	1.498	2.12	916.3	0.324	[63]
Date press cake	KOH	2633	0.952 (76.8)	0.287 (23.2)	1.239	1.88	546.8	0.208	[64]
Oily sludge + Rice husk	KOH	2575	1.080 (67.1)	0.530 (32.9)	1.610	2.50	757.6	0.294	[65]
Globe artichoke	H_3_PO_4_	2038	0.608 (24.7)	1.800 (75.3)	2.466	4.84	780.0	0.383	[19]
Rice husk	H_3_PO_4_	2028	0.787 (58.7)	0.554 (41.3)	1.341	2.64	578.1	0.285	[66]
Rawdon coal	KOH	1951	0.776 (74.0)	0.273 (26.0)	1.049	2.15	841.9	0.432	[67]
Refuse-derived fuel	KOH	1734	0.623 (53.2)	0.547 (46.8)	1.170	2.70	571.0	0.329	[68]
*Posidonia oceanica* (L.)	ZnCl_2_	1483	0.494 (48.3)	0.528 (51.7)	1.022	2.76	285.7	0.193	[55]
Commercial AC	-	1440	0.368 (51.6)	0.345 (48.4)	0.713	1.98	370.4	0.257	[18]
Pomelo skin	NaOH	1335	0.290 (37.7)	0.480 (62.3)	0.770	2.31	501.1	0.375	[69]
Vetiver roots	H_3_PO_4_	1272	0.390 (32.8)	0.800 (67.2)	1.190	3.74	394.0	0.310	[51]
Orange peel	K_2_CO_3_	1104	0.247 (40.2)	0.368 (59.8)	0.615	2.23	382.8	0.347	[54]
Waste apricot	ZnCl_2_	1060	0.150 (19.0)	0.640 (81.0)	0.790	2.98	102.0	0.096	[70]
Coffee grounds	H_3_PO_4_	925	0.211 (29.4)	0.507 (70.6)	0.718	3.10	181.8	0.197	[18]
Cotton stalk	ZnCl_2_	795	0.083 (13.2)	0.547 (86.8)	0.630	3.17	315.5	0.397	[71]
Longan seed (A6)	CO_2_	1773	0.600 (55.9)	0.474 (44.1)	1.074	2.42	1000.0	0.564	–

**Table 9:** Thermodynamic parameters of the MB adsorption on longan seed-activated carbon.

*C_e_* (mg/L)	Δ*H°*	Δ*S°*	Δ*G°* (kJ/mol)		
	(kJ/mol)	(kJ/mol-K)	308 K	318 K	328 K
20	8.03	0.0531	−8.35	−6.98	−5.61
40	9.50	0.0535	−8.84	−7.45	−6.06
80	10.98	0.0538	−9.42	−8.05	−6.69

## Data Availability

The data presented in this study will be available upon request.

## Supplementary Materials

The following are available online, Figure S1: Nitrogen adsorption isotherms of (a) microporous-activated carbon prepared by the two-step activation method and (b) mesoporous-activated carbon prepared by the OTA method, Figure S2: The determination of pH_PZC_ of activated carbon from the intersection between the curve of pH_initial_ vs. pH_final_ and the straight line of pH_initial_ = pH_final_.
