# Supplementary material for: The Use of High Surface Area Mesoporous-Activated Carbon from Longan Seed Biomass for Increasing Capacity and Kinetics of Methylene Blue Adsorption from Aqueous Solution
Source: Molecules. 2021 Oct 28;26(21):6521. doi: 10.3390/molecules26216521 (PMC8587158; doi:10.3390/molecules26216521)
Supplement: Supplementary file 1 [file molecules-26-06521-s001.zip › molecules-1430153-supplementary.pdf]

Supplementary materials

The Use of High Surface Area Mesoporous-Activated Carbon from Longan Seed Biomass for Increasing Capacity and Kinetics of Methylene Blue Adsorption from Aqueous Solution

Panuwat Lawtae and Chaiyot Tangsathitkulchai *

Institute of Engineering, School of Chemical Engineering, Suranaree University of Technology, Muang District, Nakhon Ratchasima 30000, Thailand; pnwlawtae@gmail.com

* Correspondence: chaiyot@sut.ac.th

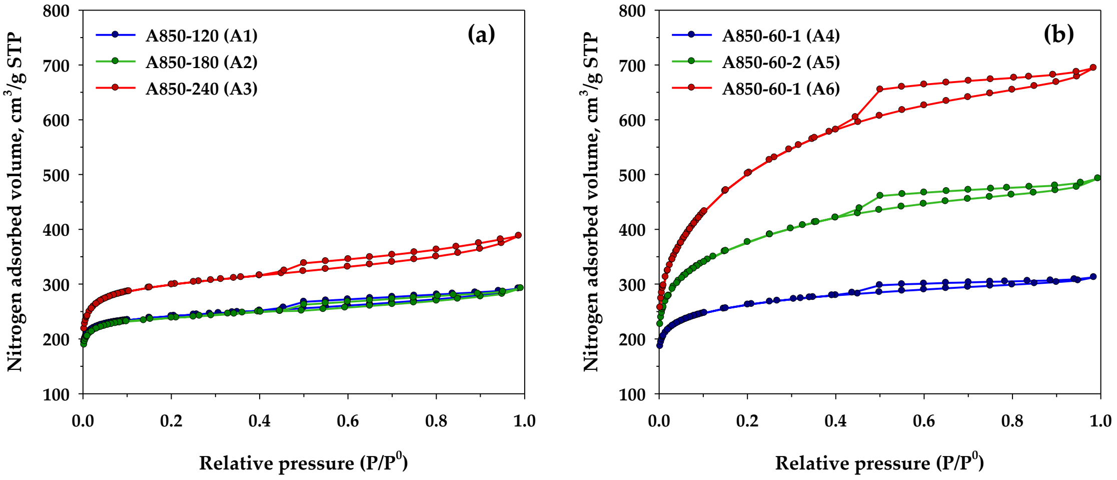

Figure S1. Nitrogen adsorption isotherms of (a) microporous activated carbon prepared by the two-step activation method and ((b) mesoporous activated carbon prepared by the OTA method.

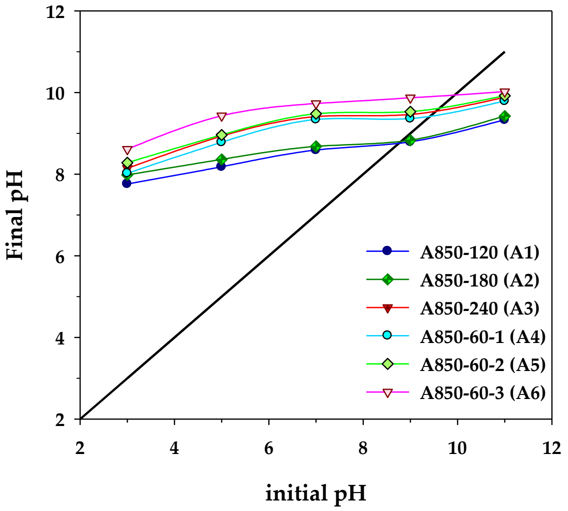

Figure S2. The determination of pH_{PZC} of activated carbon from the intersection between the curve of $pH_{initial}$ vs pH_{final} and the straight line of $pH_{initial} = pH_{final}$.
